# Using re-randomisation to improve patient recruitment and increase statistical power in clinical trials

**DOI:** 10.1186/1745-6215-16-S2-P219

**Published:** 2015-11-16

**Authors:** Brennan Kahan, Andrew Forbes, Caroline Dore, Tim Morris

**Affiliations:** 1 Pragmatic Clinical Trials Unit, Queen Mary University of London, London, UK; 2 School of Public Health and Preventive Medicine, Monash University, Melbourne, Australia; 3 Comprehensive Clinical Trials Unit, University College London, London, UK; 4 Hub for Trials Methodology Research, MRC Clinical Trials Unit at UCL, London, UK

## 

Patient recruitment is a major challenge for randomised trials. Reviews of publicly funded UK trials have found that 45 to 69% fail to recruit to target. This increases costs, delays results, and adversely impacts on the feasibility of conducting trials for conditions where there is a limited patient pool.

For some conditions a patient requires treatment on multiple occasions. For example, patients with sickle cell disease require pain relief for each pain crisis, or women having fertility treatment undergo multiple treatment cycles until becoming pregnant. The current norm is for patients to be included for only one treatment episode. We propose a re-randomisation design, allowing patients to be randomised on multiple occasions, which could substantially increase the recruitment rate.

We describe some properties of the re-randomisation design, such as the conditions required to obtain unbiased estimates of the treatment effect and control type I error rates, and offer advice on practical design and analysis issues. We show that this design can reduce the required number of patients compared with a parallel group design. It can be used in a wider variety of settings than a crossover design and may be more patient-centred as the number of treatment periods is determined by participants rather than the trial design. We also highlight situations where this design would be inappropriate.

